# Supplementary material for: Identification and functional analysis of non-coding regulatory small RNA FenSr3 in Bacillus amyloliquefaciens LPB-18
Source: PeerJ. 2023 May 15;11:e15236. doi: 10.7717/peerj.15236 (PMC10194069; doi:10.7717/peerj.15236)
Supplement: Supplemental Information 4 [file peerj-11-15236-s004.zip › KO/CK-vs-T1_map/map05213.html]

KEGG PATHWAY: Endometrial cancer - Reference pathway


|  |  |
| --- | --- |
| **Endometrial cancer - Reference pathway** |  |

[
Pathway menu
| Organism menu
| Pathway entry
| Show description
| User data mapping
]

Endometrial cancer (EC) is the most common gynaecological malignancy and the fourth most common malignancy in women in the developed world after breast, colorectal and lung cancer. Two types of endometrial carcinoma are distinguished with respect to biology and clinical course. Type-I carcinoma is related to hyperestrogenism by association with endometrial hyperplasia, frequent expression of estrogen and progesterone receptors and younger age, whereas type-II carcinoma is unrelated to estrogen, associated with atrophic endometrium, frequent lack of estrogen and progesterone receptors and older age. The morphologic differences in these cancers are mirrored in their molecular genetic profile with type I showing defects in DNA-mismatch repair and mutations in PTEN, K-ras, and beta-catenin, and type II showing aneuploidy, p53 mutations, and her2/neu amplification.

|  |  |  |
| --- | --- | --- |
| Reference pathway | 184% 150% 122% 100% 82% 67% 55% | 图片下载 |
